# Arsenic trioxide as an inducer of immunogenic cell death

**DOI:** 10.1080/2162402X.2023.2174723

**Published:** 2023-02-06

**Authors:** Oliver Kepp, Hui Pan, Peng Liu, Guido Kroemer

**Affiliations:** a Metabolomics and Cell Biology Platforms, Gustave Roussy Cancer Center, Université Paris Saclay, Villejuif, France, captain.olsen@gmail.fr; b Centre de Recherche des Cordeliers, Equipe labellisée par la Ligue contre le cancer, Université Paris Cité, Sorbonne Université, Inserm U1138, Institut Universitaire de France, Paris, France; c Institut du Cancer Paris CARPEM, Department of Biology, APHP, Hôpital Européen Georges Pompidou, Paris, France

Arsenic trioxide (ATO) is often combined with all-trans retinoic acid (ATRA) to treat promyelocytic leukemia (PML) with a rather high success rate. In mice, it has been documented that ATRA is much more efficient against PML developing in immunocompetent than in immunodeficient mice,^1,2^ pleading in favor of the idea that the antileukemic action of ATRA depends on the immune system. However, no such immune-dependent effects of ATO have been described in PML. Nonetheless, it has been shown that ATO increases lymphokine activated killer (LAK)-mediated cytotoxicity against human myeloma cells^3^ and enhances the efficacy of Bacille Calmette-Guérin (BCG) immunotherapy in a mouse model of bladder cancer.^4^ Moreover, ATO has been demonstrated to deplete regulatory T cells in a mouse model of colon cancer.^5^ Of note, in a recent paper published in *Cellular and Molecular Immunology*, Chen *et al*. demonstrate that ATO can trigger immunogenic cell death (ICD) in solid tumors.^6^

The concept of ICD, initially established in cells undergoing apoptosis, has recently been extended to other variants of regulated cell death such as necroptosis, pyroptosis, and ferroptosis.^7–11^ Canonical ICD triggers the emission of a set of danger associated molecular patterns (DAMPs), which act on specific pattern recognition receptors (PRRs) expressed by antigen presenting dendritic cells (DCs), thus stimulating phagocytosis of malignant cells and antigen presentation of tumor-associated antigens by DCs.^12,13^ Mature DCs facilitate cross-presentation of tumor antigens to cytotoxic T lymphocytes (CTL) as well as the education of memory T cells, altogether conferring efficacy to cancer therapies that last beyond treatment discontinuation.^12^ Preclinical and clinical data support the notion that ICD inducers can be advantageously combined with additional immunotherapies such as immune checkpoint blockade targeting the PD-1/PD-L1 interaction.

In their work, Chen *et al*. discovered that *in vitro* cultures of malignant cells with ATO led to the generation of a whole cell vaccine that could be injected into mice to reduce cancer growth in prophylactic as well as in therapeutic settings.^6^ These anticancer effects of ATO-treated cancer cells were lost or attenuated upon depletion of CD8^+^ (but not NK1.1^+^) T cells, as well as after blocking either interferon-ϒ (IFN_ϒ_) or the Type-1 interferon receptor (IFNAR) with suitable antibodies. ATO-treated cells manifested several well-established hallmarks of ICD including the release of ATP and high-mobility group B1 (HMGB1) protein, the exposure of calreticulin (CALR) on the cell surface, the induction of cGAMP production, and the H151-repressible (and hence likely STING-dependent) induction of interferon-β1 (IFNβ1).^6^

At the mechanistic level, the authors described that ATO induced biochemical characteristics of several cellular stress and death routines including autophagy, apoptosis, ferroptosis, necroptosis, and pyroptosis that all were blunted when ATO-induced oxidative stress was quenched by N-acetyl-L-cysteine. However, the knockout or knockdown of genes required for apoptosis (*Bak, Bax*), autophagy (*Becn1*), ferroptosis (*Acsl4*), necroptosis (*Mlk1, Ripk3*) and pyroptosis (*Gsdmd, Gsdme*) did not prevent ATO-induced cell killing, indicating that none among these pathways is indispensable for the lethal outcome of ATO treatment. In stark contrast, knockout of several among these effectors, in particular *Acsl4* (involved in ferroptosis) and *Mlk1* or *Ripk3* (involved in necroptosis), fully abolished the capacity of ATO-treated TC-1 non-small cell lung cancer to induce prophylactic anti-TC-1 immune responses in mice. The knockout of *Bak* or *Bax* (both involved in apoptosis) or *Becn1* (involved in autophagy) yielded a partial phenotype (i.e., attenuation but not abolition of vaccination), while the knockout of *Gsdmd, Gsdme* (involved in pyroptosis) failed to affect the capacity of ATO-treated TC-1 cells to induce a protective anti-TC-1 immune response. Hence, several among the ATO-triggered subroutines (autophagy, apoptosis, ferroptosis, necroptosis) contribute to the vaccination effect. Mechanistically, the authors showed that *Acsl4*, Bak, Bax,*Becn1, Mkl1*, and *Ripk3* contributed to ATO-induced ATP release; *Mkl1* and *Ripk3* to HMGB1 release; *Acsl4*, Bak, Bax,*Mlkl*, and *Ripk3* to CALR exposure; and *Acsl4, Mlkl*, and *Ripk3* to extracellular cGAMP accumulation, IFNβ1 secretion and transcription of IFN-stimulated genes (ISG).^6^

In a subsequent step, Chen *et al*. showed that intratumoral injection of ATO failed to suppress the outgrowth of TC-1 or MCA205 fibrosarcomas *in vivo*.^6^ In contrast, immunization with an ATO-based whole-cell vaccine partially restrained the growth of established TC-1 or MCA205 tumors. Such therapeutic whole-cell vaccines required expression of *Acsl4, Mlkl*, and *Ripk3*. Of note, the failure of *Ripk3*^−/−^ cells to act as a therapeutic vaccine could be restored when ATO treatment *in vitro* was combined with drugs that enhance extracellular ATP (the apyrase inhibitor ARL67156), ligate Toll-like receptor-4 (monophosphoryl lipid A) or activate STING (2ʹ3’-cGAMP). Of note, the ATO-based whole-cell vaccine increased its capacity to restrain tumor growth if combined with PD-1 blockade.^6^ Although these latter effects appeared additive (rather than synergistic), they delineate a possible strategy for improving the efficacy of therapeutic vaccination with cells undergoing ICD.

Altogether, the aforementioned data support the idea that ATO, which is likely the first antineoplastic chemotherapy that was used in the world (in particular in China), can stimulate several facets of ICD. Importantly, ATO activates a mixed pattern of stress and death pathways, many of which contribute to the immunogenic effects of ATO-treated cancer cells (Figure 1). Future studies must explore the possibility that such a pleiotropic pattern of ICD might be more efficient in yielding therapeutic cancer vaccines than ICD relying on single or dual-cell stress/death subroutine(s). In other words, would a mixture of four stress/death pathways (autophagy, apoptosis, ferroptosis, and necroptosis) generate a more efficient ICD-based vaccine that only involves one or combinations of two or three pathways? The answer to this question will have implications for the future clinical development of therapeutic vaccines.

**Figure 1.**
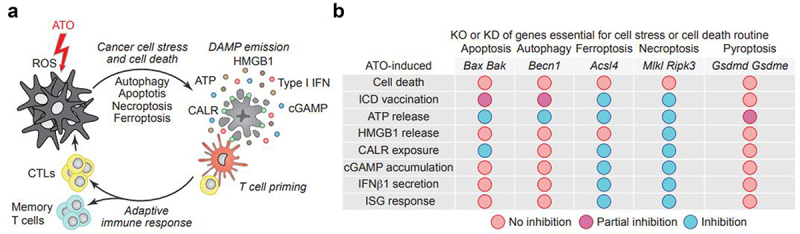
ATO induces canonical and non-canonical traits of immunogenic cell death. (a) Reactive oxygen species (ROS) induced by arsenic trioxide (ATO) trigger both canonical and non-canonical immunogenic cell death (ICD) responses such as the onset of autophagy and apoptosis as well the execution of necroptosis and ferroptosis, respectively. ATO-induced ICD facilitates the emission of danger associated molecular patterns (DAMP), including the exposure of calreticulin (CALR), the release of adenosine triphosphate (ATP), the exodus of high mobility group box 1 (HMGB1), the liberation of cyclic guanosine monophosphate–adenosine monophosphate (cGAMP) as well as Type I interferon (Type I IFN) responses. ICD-associated DAMPs act on dendritic cells (DC) and stimulate antigen presentation to T cells. T cell priming ultimately leads to clonal expansion of cytotoxic T lymphocytes (CTL) and the education of memory T cells, altogether inducing anticancer immune responses. ICD inducers can be advantageously combined with immune checkpoint blockade leveraging the full potential of T cell-mediated adaptive immunity. (b) Mechanistic effects on ICD induction of knockout (KO) or knockdown (KD) of essential genes in cell stress and cell death routines.

## Data Availability

All data that led to the conclusions in this manuscript have been included and all sources have been described.
